# Enhancement of cellular activity in hyperglycemic mice dermal wounds dressed with chitosan-alginate membranes

**DOI:** 10.1590/1414-431X20198621

**Published:** 2019-12-20

**Authors:** J.S.C. Breder, A.L.R. Pires, F.F. Azevedo, P.P. Apolinário, T. Cantaruti, S.I. Jiwani, Â.M. Moraes, S.R. Consonni, E.P. Araújo, G.G. Adams, M.J.A. Saad, M.H.M. Lima

**Affiliations:** 1 Faculdade de Enfermagem, Universidade Estadual de Campinas, Campinas, SP, Brasil; 2 Departamento de Engenharia de Materiais e Bioprocessos, Faculdade de Engenharia Química, Universidade Estadual de Campinas, Campinas, SP, Brasil; 3 Faculty of Medicine and Health Sciences, University of Nottingham, Nottingham, UK; 4 Departamento de Bioquímica e Biologia Tecidual, Instituto de Biologia, Universidade Estadual de Campinas, Campinas, SP, Brasil; 5 Departamento de Clínica Médica, Faculdade de Ciências Médicas, Universidade Estadual de Campinas, Campinas, SP, Brasil

**Keywords:** C57BL/6J mice, Wound healing, Wound dressing, Polysaccharides, Diabetes

## Abstract

The use of specially designed wound dressings could be an important alternative to facilitate the healing process of wounds in the hyperglycemic state. Biocompatible dressings combining chitosan and alginate can speed up wound healing by modulating the inflammatory phase, stimulating fibroblast proliferation, and aiding in remodeling phases. However, this biomaterial has not yet been explored in chronic and acute lesions of diabetic patients. The aim of this study was to evaluate the effect of topical treatment with a chitosan-alginate membrane on acute skin wounds of hyperglycemic mice. Diabetes mellitus was induced by streptozotocin (60 mg · kg^-1^ · day^-1^ for 5 days, intraperitoneally) and the cutaneous wound was performed by removing the epidermis using a surgical punch. The results showed that after 10 days of treatment the chitosan and alginate membrane (CAM) group exhibited better organization of collagen fibers. High concentrations of interleukin (IL)-1α, IL-1β, granulocyte colony-stimulating factor (G-CSF), and tumor necrosis factor-alpha (TNF-α) were detected in the first and second days of treatment. G-CSF and TNF-α level decreased after 5 days, as well as the concentrations of TNF-α and IL-10 compared with the control group (CG). In this study, the inflammatory phase of cutaneous lesions of hyperglycemic mice was modulated by the use of CAM, mostly regarding the cytokines IL-1α, IL-1β, TNF-α, G-CSF, and IL-10, resulting in better collagen III deposition. However, further studies are needed to better understand the healing stages associated with CAM use.

## Introduction

Wound healing is a complex process, being essential for skin repair. It is basically comprised of three phases: inflammation, proliferation, and maturation, which overlap in time and space (1,2). Many cell types may be involved in this process, and phenomena as the influx of macrophages, neutrophils, endothelial cells, platelets, keratinocytes, and fibroblasts, as well as the release of cytokines and growth factors, are commonly observed during wound-healing phases (1,2).

Cytokines such as interleukin-1 alpha (IL-1α), IL-1β, and tumor necrosis factor-alpha (TNF-α) have a key role in the wound healing process (1,2). These proteins trigger or enhance inflammation (2). IL-1β and TNF-α, for instance, are produced by macrophages, keratinocytes, vascular endothelial cells, fibroblasts, monocytes, and T lymphocytes in wounds (3). Another factor present in the inflammatory phase of skin lesions is the granulocyte colony stimulating factor (G-CSF), which has the ability to accelerate hematopoiesis in the bone marrow and make these cells available in the bloodstream. Consequently, myeloid cells such as mature neutrophils are released into the peripheral circulation. Treatment for 4 to 5 days of G-CSF mice results in an increase in the number of circulating neutrophils, probably due to increased granulopoiesis and to an indirect effect of G-CSF on neutrophil mobilization (4,5). Consequently, more mature neutrophils are developed and released into peripheral circulation. Chemoattractant substances establish gradients that guide neutrophils and other types of leukocytes into the wound sites (6).

In addition, inflammatory and anti-inflammatory cytokines are essential regulators of wound repair and their balance is fundamental for optimum wound closure. The role of IL-10 is to limit and terminate inflammatory responses. Moreover, it regulates growth and/or differentiation of various immune cells, keratinocytes, and endothelial cells.

Several factors can interfere in one or more phases of this process and affect wound healing. When associated with diabetes types 1 and 2 for long periods, hyperglycemia affects the wound healing process through one or more biological mechanisms, by giving rise to exacerbated and prolonged cutaneous inflammation, micro- and macro-circulatory dysfunction (7,8), and impaired neuropeptide signaling (9). These effects result in decreased release of cytokines and growth factors and poor oxygen supply to the wound (10,11), and these factors also contribute to slow wound repair (10).

The search for wound dressings capable of simultaneously protecting the wound and contributing to healing modulation is, therefore, highly relevant for patients with diabetes, which sometimes develop a hyperglycemic state. The use of natural polymers in dressing materials introduces important advantages, such as improved biocompatibility and biodegradability, non-toxicity, safety, similarity to the extracellular matrix, as well as the ability to induce and stimulate wound healing (12). Chitosan and alginate are biopolymers widely used in the production of wound dressings.

A clinical study that compared the use of chitosan wound dressing to a traditional Vaseline gauze (control) in patients with unhealed chronic wounds, including diabetic foot ulcers, showed promising results (13). The authors noticed wound reepithelialization after 4 weeks of treatment and patients reported lower pain levels when chitosan was used. Chitosan consists of N-acetylglucosamine and N-glucosamine units and, according to Karri et al. (14), chitosan can depolymerize and release N-acetylglucosamine, which initiates fibroblast proliferation and helps in ordered collagen deposition.

Alginate also shows a positive influence in diabetic wound healing. Its injection in mice lesions is capable of reducing the wound size by about 50% ten days after the first application (15). When calcium alginate dressings contact exudative lesions, the calcium ions are exchanged with sodium ions present in the exudate, resulting in the formation of a sodium-alginate gel. This gel keeps the lesion moist, promotes tissue granulation, and accelerates epithelization, consequently improving the healing process (16).

When combined, chitosan and alginate may form a stable polyelectrolyte complex (PEC) that results in relatively transparent film capable of swelling in the presence of body fluids, such as exudates, with appropriate mechanical properties (17,18). Chitosan-alginate membranes are capable of modulating the inflammatory phase, stimulating the proliferation of fibroblasts and the production of collagen fibers, accelerating cutaneous wound healing in the earlier phases, and improving the quality of the scar tissue (17). These findings strongly support the hypothesis that chitosan-alginate membranes could exert beneficial effects on the healing process of wounds in patients with diabetes with lesions of both acute and chronic origins.

Since no reports have been found with a detailed analysis of the use of dense chitosan-alginate PEC films to treat skin lesions of hyperglycemic animals, the aim of this work was to evaluate the efficacy of chitosan and alginate membrane (CAM) dressings on skin wounds of diabetes animal models. The results showed that CAM modulated the cytokines IL-1α, IL-1β, TNF-α, G-CSF, and IL-10, resulting in better collagen III deposition in cutaneous lesions of mice.

## Material and Methods

### Animals

The animals (eight-week-old male C57BL/6J mice) were kept under specific pathogen-free conditions in individual cages maintained at 21°C, in light-controlled conditions (12-12 h light and dark cycles), and received a standard solid diet and water *ad libitum*.

### Chitosan-alginate membranes

**Figure 1:**
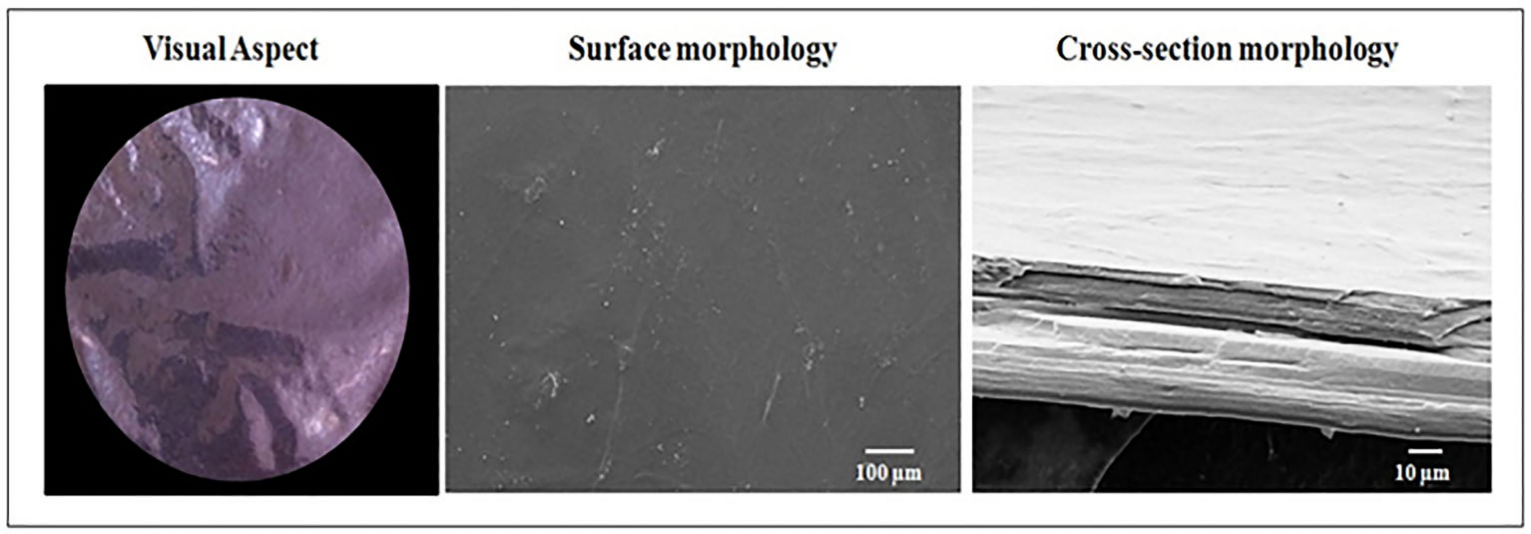
Visual aspect and morphological characteristics of chitosan-alginate membranes.

The membranes were prepared as reported by Pires and Moraes (19). Briefly, a chitosan (lot #109K0043V, 96% deacetylation degree, Sigma-Aldrich, Germany) solution at 1% (w/v) (180 mL, in 2% v/v acetic acid) was mixed with 0.5% sodium alginate (medium viscosity from *Macrocystis pyrifera*, Sigma-Aldrich, lot #058K0126) aqueous solution (360 mL) at 25°C under stirring first at 500 rpm. After being mixed, the material was further homogenized at 1000 rpm for 10 min. Then, 26.0 mL of 2 mol/L NaOH aqueous solution and 7.2 mL of 2% (w/v) CaCl_2_ aqueous solution were added to the suspension. After deaeration, the mixture was transferred to four polystyrene Petri dishes (15 cm in diameter) and dried at 60°C for 6 h. Then, the membranes were immersed in 150 mL of 2% (w/v) CaCl_2_ aqueous solution for 30 min and washed twice with 200 mL of deionized water. Next, the biomaterial was dried at room temperature for 24 h and sterilized by exposure to ethylene oxide at Acecil Central de Esterilização Comércio e Indústria (Brazil). The morphology of the membranes was evaluated by scanning electron microscopy (SEM), using a LEO 440 microscope (LEO Electron Microscopy Ltd., UK) operating at 10 kV and 50 pA. The samples were first dried, attached to a suitable metallic support, and coated with a 92 Å-thick gold layer (mini Sputter coater, SC 7620, VG Microtech, UK). Samples fractured with liquid nitrogen before metallization were used to obtain cross-section images. The membrane properties are summarized in Table 1 and their typical aspect is illustrated in Figure 1.

**Table 1:** Physicochemical characteristics of the chitosan-alginate membranes.

Property	Mean ± Standard Deviation
Thickness	
Dry membrane (mm)	0.046±0.005
Membranes after exposure to saline solution at 37°C for 24 h (mm)	0.300±0.001
Swelling in saline solution (after 24 h at 37°C) (g/g)	14±1
Mass loss in saline solution (after 7 days 37°C) (%)	12±1
Tensile strength (Mpa)	30±2
Elongation at break (%)	3.0±0.8

### Induction of diabetes mellitus

Diabetes mellitus was induced by streptozotocin (60 mg · kg^-1^ · days^-1^ for 5 days, intraperitoneally) (Sigma^®^, USA) as reported previously by Yu et al. (20). Two weeks after induction, blood was drawn from the animal tail veins and glucose concentration was analyzed using an Accu-Chek Active glucometer (Roche, Switzerland). Animals with blood glucose concentrations above 250 mg/dL were considered diabetic and selected for the study.

The Ethics Committee of the University of Campinas approved all experiments (Protocol number: 3950-1/2015). The cutaneous wound was performed on the same day of diabetes confirmation. For surgical intervention, the mice were anesthetized with intraperitoneal ketamine hydrochloride (180 mg/kg body weight) and xylazine hydrochloride (8 mg/kg body weight) (21). The animals were positioned in dorsal decubitus, the dorsal area was shaved and rinsed with alcohol 70%, and two circular wounds were produced by full epidermis removal using a 6.0 mm punch (Miltex^®^ Inc., USA). Previously hydrated chitosan-alginate membranes and 0.9% (w/v) NaCl aqueous solution were placed on each dorsal excision site. After the wound procedure, the animals received 20 mg/kg of intraperitoneal tramadol chlorhydrate (Medley Pharmaceutical Industry, Brazil), once a day for two days after wound induction (22).

The animals were divided into two groups and placed individually in numbered cages: the CAM group, which was treated with the chitosan-alginate membranes (circular shape, 6 mm in diameter) previously moistened with 0.9% (w/v) NaCl aqueous solution; and the control group (CG), which was treated only with 0.9% saline (500 μL per lesion). The wounds of all animals were hydrated daily with 0.9% saline (500 μL) until the 10th day of follow-up. The chitosan-alginate membranes were not removed during the treatment.

### Wound healing assessment

To evaluate wound closure, the lesions were photographed daily by the same examiner with a Canon^®^ Power Shot digital camera (model SX400 IS, Japan). A tripod was used to maintain the same camera-to-wound distance in all experiments. The wound area was measured using the ImageJ software (National Institutes of Health, USA). Wound closure (Wc) was defined as the reduction of wound area and the results are reported as percentage of the original lesion area, as follows: Wc = d_t_ / d_0_ × 100, where d_0_ refers to the total lesion area immediately after wounding and d_t_ to the wound area still uncovered with epidermis at time t.

The total wound length was defined as the distance between opposite wound edges. The wound edge was determined based on the typical features of normal skin and newly formed dermal tissue. Since the chitosan-alginate membrane is transparent, it was possible to monitor the wound aspect and size without removing it.

### Tissue processing and staining

After anesthetizing the mice with intraperitoneal ketamine hydrochloride (180 mg/kg body weight) and xylazine hydrochloride (8 mg/kg body weight) (21), the fragments of skin of all groups were collected at days 1, 2, 5, and 10 post-lesion. After the surgical procedure, all animals were euthanized with an overdose of anesthetic, in accordance with ethical standards. The dissected skin tissues were fixed by immersion in paraformaldehyde in distilled water (4%), dehydrated in increasing concentrations of ethanol, followed by immersion in xylol and embedded in paraffin. The blocks were sectioned at 5.0 µm and placed on microscope slides. The wound sections were stained with hematoxylin and eosin. Digital images were captured under an Olympus (Japan) photomicroscope (model U-LH100HG), and representative images of the histological structures were recorded using a digital image capture and analysis system (Olympus camera, model U-TVO.63XC / T2).

The histological sections were stained using the 1% Picrosirius technique (23) and analyzed under polarized light to evaluate types I and III collagen fibers. Fibers were identified by their birefringence pattern (type I: red, orange, and yellow and type III: green). The density of types I and III collagen fibers (per square micrometer) was determined using the Image Pro Plus version 6.0 (Media Cybernetics, LP, USA).

### Morphometric analysis

The number of leukocytes, fibroblasts, and new vessels and capillaries was determined in the injured area 5 and 10 days after wounding at 100× magnification. Fifteen areas of 100 μm were analyzed in each region. The areas analyzed were determined by following a straight line, always moving the field in a single direction, with spacing of 100 to 300 μm between two regions analyzed depending on the characteristic of the repair region, keeping five areas below the injured epidermis, five in a lower region, and five areas close to the flesh panniculus (24). This morphometric analysis was performed by blinded raters.

### Cytokine contents in wound tissue

Wound lesion samples collected at 1, 2, 5, and 10 days after wounding were immediately packed in dry ice and kept frozen (−80°C). The concentrations of IL-1α, IL-1β, G-CSF, TNF-α, and IL-10 cytokines were measured in scar tissue homogenates using Bio-plex kit according to the manufacturer's instructions (Bio-plex Pro Assays, Bio-Rad Laboratories, Life Science Group, USA) and normalized by protein concentration determined by the Bradford method (25).

### Statistical analysis

The results are reported as means±SE. Student's *t*-test for independent samples was used to compare the mean values of the cytokine contents in wound tissue and for the analysis of type I and III collagen fibers in both groups. Two-way analysis of variance (ANOVA) was used to compare the mean wound area values from days 0 to 10 after injury. In all cases, the level of significance considered enough to reject the null hypothesis was 5% (P<0.05). Data were analyzed using the GraphPad Prism 7.

## Results

**Figure 2:**
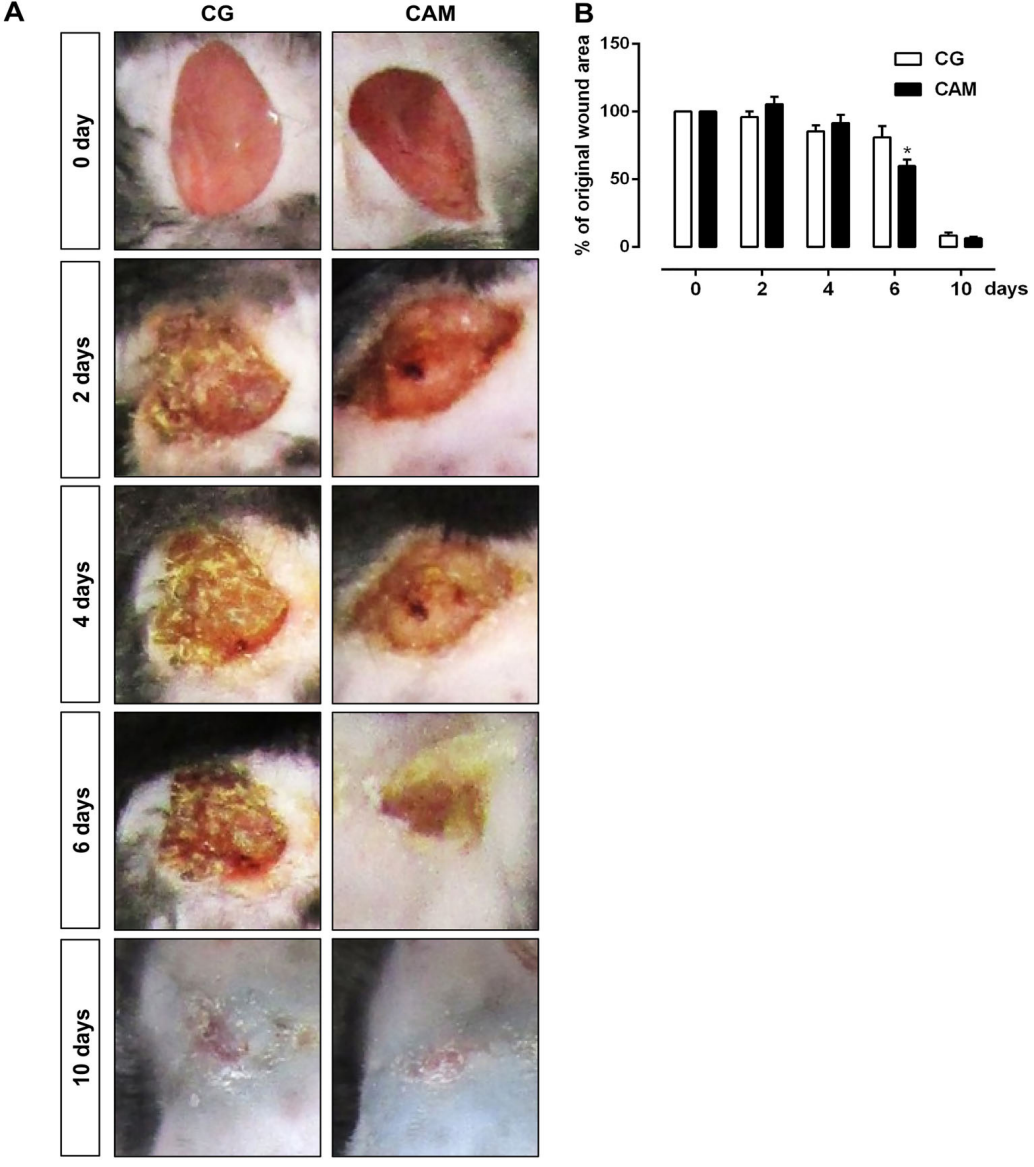
Macroscopic changes. **A**, Macroscopic changes in skin excisional wounds during wound closure in the control (CG) and chitosan and alginate membrane (CAM) groups, 0, 2, 4, 6, and 10 days post-wounding. **B**, Percentage of initial lesion area in the CG and CAM groups. Data are reported as means±SE of 5 animals per group. *P<0.05 *vs* CG (ANOVA).

**Figure 3:**
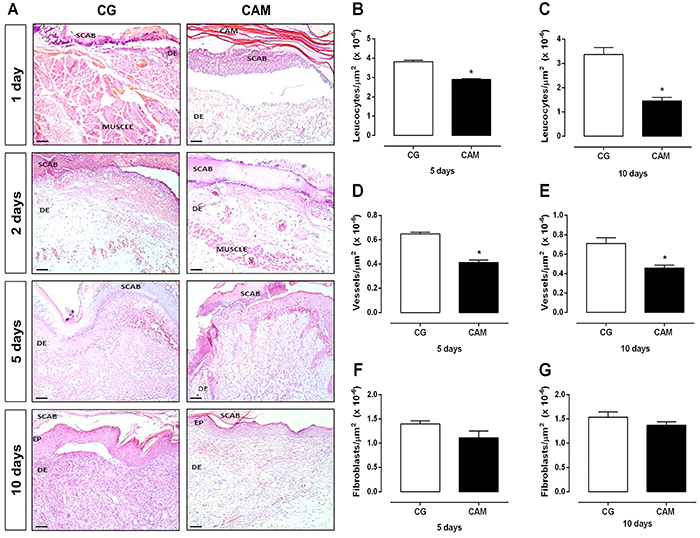
**A**, Microscopic aspect of skin excisional wounds in the control (CG) and chitosan and alginate membrane (CAM) groups, 0, 2, 4, 6, and 10 days post-wounding. Sections were stained with hematoxylin and eosin, and the images were acquired under a final magnification of 10× (scale bars: 100 µm). DE: dermis; EP: epidermis; SCAB: dry scab. Quantification of leucocytes (**B** and **C**), vessels (**D** and **E**), and fibroblasts (**F** and **G)** on days 5 and 10 post-wounding. Data are reported as means±SE of 5 animals per group. *P<0.05 *vs* CG (Student's *t*-test).

Data from macroscopic analysis of the wound area from 0 to 10 days after injury indicated that the CAM group showed a smaller wound area on the 6th day (P<0.05) compared to the CG (Figure 2A and B). According to the microscopic qualitative analysis (histological sections stained with hematoxylin and eosin), on day 10 after lesioning, the CAM group had lower inflammatory infiltrate (Figure 3C), as well as more organized dermis connective tissue (Figure 3). In addition, all animals in the CAM group (n=5) had complete reepithelization.

Morphometric analyses performed on the 5th and 10th days after the lesion indicated lower numbers of leukocytes (Figure 3B and C), blood vessels (Figure 3D and E) (P<0.05), and fibroblasts (Figure 3F and G) in the CAM group compared to the CG.

**Figure 4:**
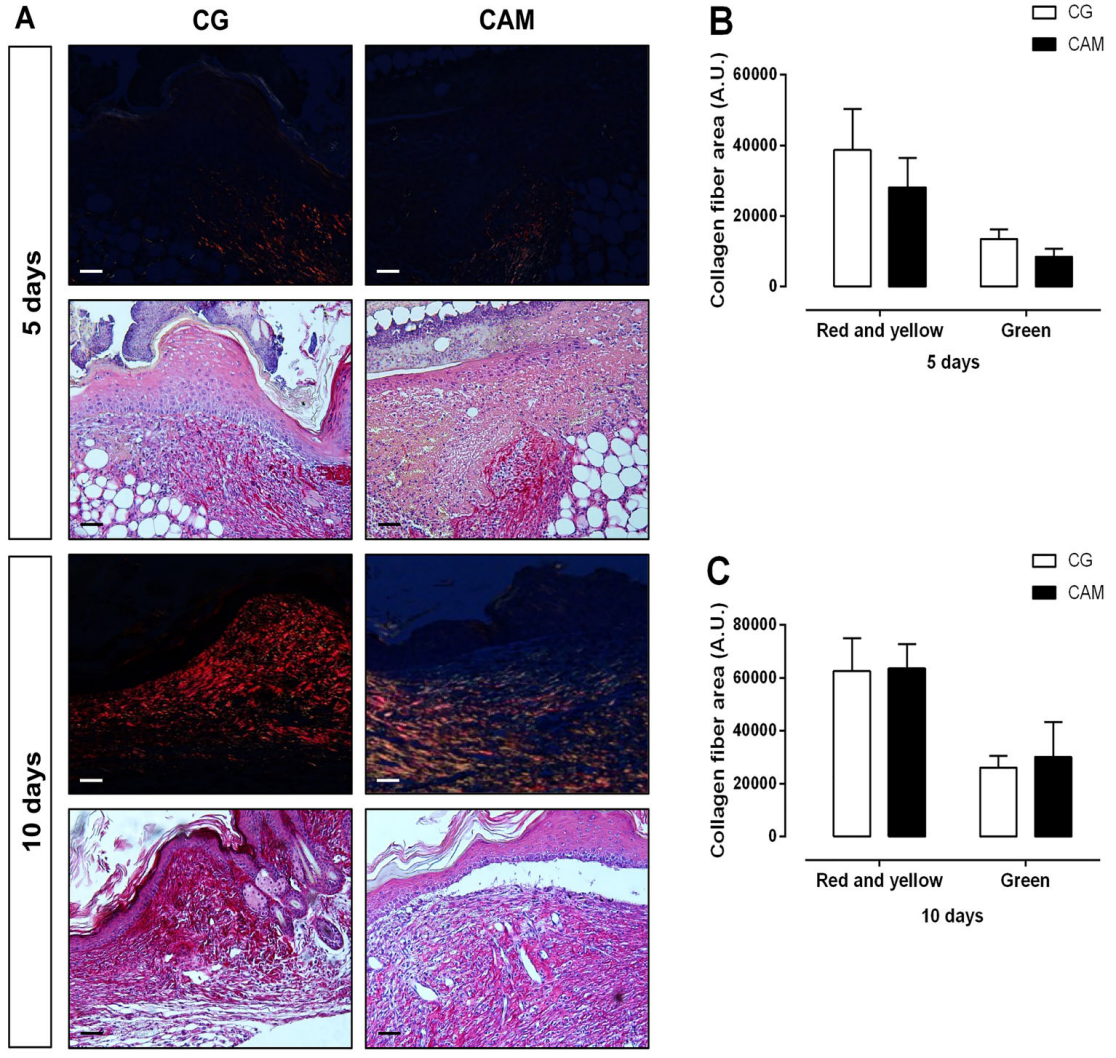
**A**, Picrosirius red-stained sections of wounds under polarized light followed by sections without polarization of control (CG) and chitosan and alginate membrane (CAM) groups, 5 and 10 days post-wounding. **B** and **C**, Analysis of collagen fibers (red and yellow and green) area of CG and CAM groups on days 5 and 10 post-wounding. The images were acquired under a final magnification of 10× (scale bars: 100 µm). The data are reported as means±SE of 5 animals per group (Student's *t*-test).

In the qualitative analysis performed on the 10th day, the CAM group showed a more regular and uniform arrangement of type I and III collagen fibers compared to the CG (Figure 4A), but there was no statistical difference in the quantitative analysis (Figure 4B and C).

**Figure 5:**
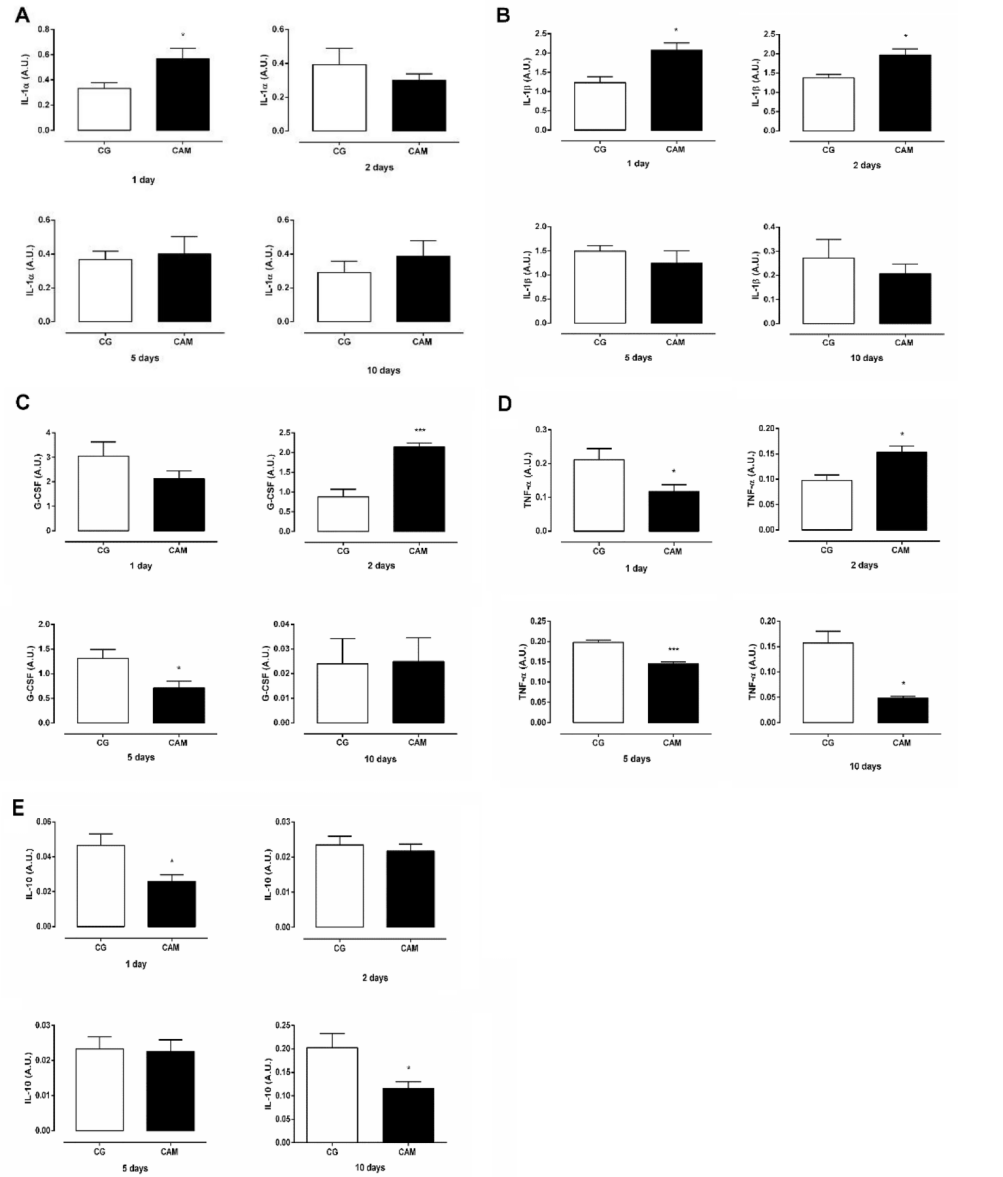
Production of cytokines during wound healing. Concentrations of **A**) interleukin-1alpha (IL-1α), **B**) IL-1β, **C**) granulocyte colony stimulating factor (G-CSF), **D**) tumor necrosis factor-alpha (TNF-α), and **E**) IL-10 in wound tissue of control (CG) and chitosan and alginate membrane (CAM) groups on days 1, 2, 5, and 10 post-wounding. Data are reported as means±SE of 5 animals per group. *P<0.05, ***P<0.001 *vs* CG (Student's *t*-test).

The multiplex assays used to determine cytokine levels in the wound area showed that IL-1α (P=0.036) (Figure 5A) and IL-1β (P=0.012) (Figure 5B) were higher in the CAM group compared to the CG on day 1. TNF-α (P=0.044) and IL-10 (P=0.028) were lower in the CAM group compared to the CG on day 1. On day 2, the CAM group had higher concentrations of IL-1β (P=0.013), G-CSF (P=0.0002), and TNF-α (P=0.014) (Figure 5B, C and D). On day 5 post-injury, G-CSF (P=0.038) and TNF-α (P=0.0003) concentrations were lower in the CAM group compared with the CG (Figure 5C and D). After 10 days, TNF-α (P=0.015) (Figure 5D) and IL-10 (P=0.027) (Figure 5E) concentrations were lower in the CAM group compared with the CG.

## Discussion

Polysaccharide materials have been used in wound healing over many years (12,17,18,26,27). Even though the wound healing process mediated by the use of these biomaterials is not yet fully understood, it is well-known that an ideal wound dressing should provide a moist environment, protect the wound from extraneous agents, absorb excess exudates, allow gas exchange, be comfortable, biocompatible, and aid in tissue regeneration (28).

Recently, it has been reported that CAMs modulate the inflammatory phase and stimulate fibroplasia and collagen fibers, accelerating the wound healing process in healthy rats (17). Positive results for wound healing in a juvenile dermatomyositis patient was also reported with the use of CAMs associated with fibrin glue and autologous fibroblasts and keratinocytes (29). According to the SEM analysis, the membrane shows a lamellar and homogeneous structure, as also reported by Zorzi Bueno and Moraes (30) and Pires and Moraes (19). The SEM analysis also showed that no pores are found on the membrane surface at the microscopic scale, indicating that it could provide appropriate protection to the wound against the permeation of contaminants from the environment, a property further reinforced by its fibrous structure (12,17,19).

In addition, the membrane is transparent (Figure 1 and 2A), especially when wet, allowing lesion inspection during treatment without the need to remove it. The membrane is also very thin, even after swelling due to the absorption of fluids (Table 1), being potentially comfortable to the patient. According to Ma et al. (31), artificial skin substitutes should be thinner than the human dermis (0.5 to 2 mm). Furthermore, the membrane formulation used is resistant enough to be handled at the patient's bedside, and despite being capable of absorbing up to 14 g/g of saline solution, its structure is sufficiently stable in this solution for at least a week, with a mass loss of only 12%. This result was corroborated *in vivo*, since the membrane remained intact along the study, while allowing the removal of excess wound exudate. The mechanical properties of the membrane were considered to be adequate regarding tensile strength; however, the biomaterial used shows low elongation at break (3%) when dry, which limits its use to non-articulated body regions. Moist membranes, on the other hand, have significantly improved elongation (18). The membrane used in this work fulfills, then, many requirements of an ideal skin wound dressing, being also easily maintained on the lesion site after being placed over the lesions.

Deficient wound healing is observed in patients with diabetes mellitus with excessive or uncontrolled inflammation (32). Several reasons can be attributed to the delay in re-epithelialization in diabetes mellitus including various types of cells and molecular effectors, which become destructive and promote apoptosis in the diabetic wound bed (33).

To evaluate the effect of CAM-mediated therapy on the wound healing process, we first observed the rate of wound closure and compared it with the results for CG at different time points throughout the 10-day treatment period. The tissue repair of the injured area of the hyperglycemic animals evolved very similarly to the CAM group, with complete reepithelialization 10 days after injury. On the other hand, there was a decrease in inflammatory infiltrate cells in the CAM group compared with the CG, which may have contributed to the difference in wound contraction 6 days after injury.

In the qualitative analysis of collagen staining by Picrosirius, an increase of well-organized collagen fibers was observed in the CAM group. Typically, in a normal process of skin repair, type III collagen deposition is observed soon after injury, which is replaced by type I collagen in the final phase of repair (34).

The healing process is regulated by a complex signaling network involving various chemokines, cytokines, and growth factors. In this study, the CAMs modulated the increase in the expression of the inflammatory cytokines, such as IL-1β and IL-1α. These cytokines modulate the activity of various types of cells implicated in tissue repair. In diabetics with wounds, granulation tissue formation is seriously impaired because of the reduced proliferation of fibroblasts, lower levels of collagen deposition, and wound contraction (35,36). However, the literature shows that the healing process is characterized by a low-grade inflammatory state in the case of hyperglycemia associated to formation of advanced glycation products (AGEs) (11). Occurrence of hyperglycemia in the animals in this study may have contributed to a lower expression of the proinflammatory cytokine TNF-α at day 1 after wounding.

In spite of this, forty-eight hours after membrane application, an increase of TNF-α and G-CSF concentrations was observed. TNF-α is a pleiotropic cytokine, with the ability to affect almost every tissue and organ. Also, this cytokine is chemotactic for macrophages and participates in macrophage activation and angiogenesis stimulation (36). In addition, TNF-α promotes choroidal neovascularization by upregulating the vascular endothelial growth factor expression in retinal pigment epithelial cells through reactive oxygen species-dependent β-catenin activation and fibroblast proliferation (37).

G-CSF is a hematopoietic cytokine produced by monocytes, fibroblasts, and endothelial cells. Furthermore, bone marrow-derived stem/progenitor cells could be strongly mobilized into circulation by G-CSF (38). Five days after wounding, there was a decrease in the level of G-CSF and TNF-α. These results seem to indicate a contribution to maintaining inflammation in a well-controlled state, indicating that CAM may be a promising therapy for wounds occurring in patients with diabetes.

IL-10 is an anti-inflammatory cytokine with a significant impact on healing and epithelialization. Its overexpression decreases inflammatory mediators and promotes regenerative healing in an adult model of scar formation (39). We detected that this cytokine level was decreased in the CAM group 10 days after wounding. Interestingly, TNF-α was not detected in the animals treated with CAM in the same period, but it was observed in the CG, indicating that there is a synergistic relationship between these two cytokines. These results suggested that treatment with CAM initially upregulated the expression of pro-inflammatory IL-1β, IL1α, and TNF-α cytokines, and that the anti-inflammatory IL-10 cytokines contributed to mediate the transition between phases of wound healing.

Thus, lesion healing of diabetics is a complex process and the interaction between chemokines, cytokines, and growth factors will determine the process evolution. In this study, the inflammatory phase of cutaneous lesions of hyperglycemic mice was modulated by the use of CAM, mostly regarding the cytokines IL-1α, IL-1β, TNF-α, G-CSF, and IL-10, resulting in better collagen III deposition. However, further studies are needed to better understand the healing stages associated with CAM use.
